# Application of an Enhanced Version of Recursive Operability Analysis for Combustible Dusts Risk Assessment

**DOI:** 10.3390/ijerph17093078

**Published:** 2020-04-28

**Authors:** Marco Barozzi, Sabrina Copelli, Martina Silvia Scotton, Vincenzo Torretta

**Affiliations:** 1 Department of Science and High Technology, Università degli Studi dell’Insubria, via Vico, 46, 21100 Varese, Italy; marco.barozzi89@gmail.com (M.B.); sabrina.copelli@uninsubria.it (S.C.); martina.scotton@gmail.com (M.S.S.); 2 Department of Theoretical and Applied Sciences, Università degli Studi dell’Insubria, via Vico, 46, 21100 Varese, Italy

**Keywords:** recursive operability analysis, dust explosion, safety engineering, risk assessment, fault tree analysis

## Abstract

Organic dust explosions were and are still today a critical issue in the food, pharmaceutical, and fine chemical industry. Materials such as flour, corn starch, sugar and APIs represent a cause of severe accidents. In this framework, we investigated a modified version of Recursive Operability Analysis−Incidental Sequence Diagrams (ROA–ISD), called ROA Plus−ISD, specifically tailored to describe industrial processes involving organic combustible dusts. Compared to more classical techniques such as Hazard and Operability (HazOp), ROA−ISD allows for a direct generation of fault trees, providing a useful tool to connect Qualitative with Quantitative Risk Analysis (QRA). ROA Plus−ISD is very similar to ROA−Cause Consequence Diagrams (CCD), which has already proven to be an effective tool to perform both risk assessment on existing plants and reconstructing already occurred accidents, given its logical structure and width of the application fields. In this work, we modified specific parts of the standard ROA−CCD method: (1) the Failure Mode and Operability Analysis (FMEA) database has been structured in order to retrieve the well-known explosion pentagon (for dusts) and all the instruments, devices, apparatuses and controllers typical of industries which process organic dusts; (2) a new comprehensive list of process variables has been compiled. In this way, it is possible to tailor the information required for the generation of the fault trees concerning top events involving mainly dust explosions and fires. This method has been implemented in order to reconstruct the dynamics of the February 2008 Imperial Sugar refinery plant accident (Port Wentworth, GA, USA). Results demonstrated the applicability of the enhanced method by highlighting the criticalities of the process already showed by a previously detailed reconstruction performed by the Chemical Safety Board.

## 1. Introduction

Industrial safety has been always considered of great interest and importance worldwide [1,2]. Particularly, in the process industry field, many efforts have been made in both legislation and implementation of safety systems/barriers to promote the mitigation of fatal accidents, such as fires, explosions and toxic releases. Risk mitigation in production facilities is still a matter of great interest, especially in activities that represent a serious hazard to human health, environment, and industrial plants.

In the last decades, Quantitative Risk Analysis (QRA) [3,4] has become established as a paramount method to perform a risk assessment, given the possibility to associate an objective quantity (such as a probability) to an unwanted event. However, QRA is a general concept, and it does not automatically provide a specific technique or procedure to be followed. Usually, the analysis consists in several steps, including identification of accidental scenarios and estimation of both magnitude and probability of occurrence of undesired events [5]. Historically, several methods were proposed in order to perform better QRA. For what concerns the identification of an accidental scenario, the Hazard and Operability (HazOp) technique, introduced in 1983 by Kletz [6], was developed in order to classify process criticalities for the chemical industry, working around process variable deviations. Nowadays, it is still potentially the most used risk analysis method inside chemical companies. The HazOp method excels in identifying accidental scenarios and critical parts of the plant, and, given its well-defined scheme, it provides objective results. However, the information recorded from an HazOp analysis is a not structured list of causes and consequences of process variables deviations. For this reason, it is not a good tool to estimate accident probabilities, which is a core part of the risk index definition. Other methods have been proposed and developed, with the aim of improving risk estimation. The Failure Mode and Operability Analysis (FMEA), or the Failure Mode and Criticality Operability Analysis (FMECA) [5,7], are techniques dedicated to the identification of accidental scenarios based upon the failure modes of components present in process equipment, such as valves and switches. FMECA implements a criticality index, in addition to the classic FMEA, highlighting only the most crucial components of the plant. Hence, FMEA/FMECA, is a good tool to find the components involved in potential accidents, and usually, the associated failure rates can be recovered from a literature database. However, it still provides unstructured information, leaving unknown an accidental scenario probability estimation. For the estimation of probabilities, which are required for the risk index assessment, Fault Tree Analysis (FTA) [8,9] is among the most used methods, given its high flexibility in the application field. FTA requires a combination of basic events connected through logic gates. This task can be quite complicated to be performed, especially inside complex plants with a lot of human interactions with the system. It is clear then, that a risk assessment can be effective if it is obtained through the synergy of many methods, each one dedicated to describing specific risk analysis steps.

In this framework, several integrated methods have been thoroughly developed and presented, such as MAXCRED [10], SCAP [11]. The purpose of such methods is to both identify likely accidental scenarios in chemical industries, such as fires and explosions, addressing the magnitude of accident consequences through simulations, establishing individual, plant and social risk factors. MAXCRED is dedicated to consequences estimation, while SCAP is devoted to safety management, and it is applied to define and estimate the effect of risk mitigation means. 

Dynamic models have been also developed, like the bow-tie approach proposed by Khazad et al. [12]. Such models are very useful to keep an updated probability estimation for Top Events, in highly dynamic systems.

One of the limitations of such methods lies in a lack of generality in the identification of the mechanics of industrial accidents: since they try to embrace a vast amount of chemical processes, the risk assessment needs to be executed by a highly skilled project team, which must be able to identify the criticalities of the involved issues and translate them in a Fault Tree Analysis.

Historically, a first attempt to integrate an HazOp to FTA method has been the introduction of the Recursive Operability Analysis (ROA), proposed by Piccinini and Ciarambino [13,14]: the main concept consists in working around process variables deviations (following the same pattern of HazOp) but, instead of simply listing them, it interconnects them, based upon a cause-sequence criterion. The method was efficiently used to perform risk assessments in the chemical industry [15,16], and it was also improved. However, ROA presents some flaws: it does not consider that plant state according to protection means working properly, and it can be time expensive, requiring several records even for relatively simple systems [17]. The original ROA has been recently updated, with the implementation of the ROA−Cause Consequence Diagrams (CCD) technique [17,18]. This method, through a well-structured ROA table (that is very similar to an HazOp analysis), it is possible to deduce a complete fault tree, either including the failure of both automatic and manual protection devices or considering the plant state assuming that protections worked properly. The method is relatively fast to be executed in comparison with the original ROA, as shown in the work of Contini et al. [17]. However, since the ROA-CCD it is still an HazOp-based model, it does not hint which process variable deviations will contribute to accidental Top Events. For these reasons, this paper is devoted to the formulation of a method, with the same structure of the ROA−CCD, that can almost automatically generate and resolve a fault tree, starting from basic process information. Unfortunately, it is unlikely to produce a method applicable to all chemical plants, given the width of potential accidents. The proposed method, which will be referred to as Recursive Operability Analysis Plus with Incidental Sequence Diagrams (ROA Plus−ISD) is a technique dedicated to identify accidental scenarios and their probability estimation, and it is tailored to specifically treat dust explosions. Dust explosions are fatal and severe accidents that affect process industries [19], and methods to carefully establish the risk associated with explosive dust are subject of great interest [20]. When talking about process variables and dust explosions, it is possible to find conjunction in the well-known dust explosion pentagon [21]. According to the pentagon, five conditions have to be simultaneously satisfied in order to make possible a dust explosion: the presence of combustible dust, an oxidizing agent (usually oxygen), dispersion in air, confinement and an ignition source.

The structure of the ROA Plus−ISD consists of defining a priori all process variables, human operations and components involved, moving around the conditions represented by the dust explosion pentagon. Basic events which will be required for the computation of the FTs are deduced by performing an FMEA. Once all the required elements are defined, it is possible to generate the ROA table, where only the deviations related to dust explosion must be considered. Afterward, starting from every single record of the ROA table (that is, a single row), the corresponding ISD can be generated and, by repeating the procedure on all records, the final Fault Trees for the identified Top Events are automatically generated, by matching all the correlated ISDs. Finally, FTA can be performed with dedicated software, such as PROFAT [22], OpenFTA, FaultTree+ [9] or ASTRA [23].

The main difference with respect to ROA-CCD consists in a return to the use of the original Incidental Sequence Diagrams (ISDs) for the generation of fault trees, maintaining the structure of the ROA-CCD table. 

This new technique was applied to a case study: the risk assessment of the sugar conveyance line of the former Imperial Sugar plant (Port Wentworth, GA, USA). This site suffered a severe dust explosion throughout the plant in February 2008, and the main cause was the implementation of a closed metal barrier on the conveyor belts, which eventually led to the primary explosion. Risk assessment of this part of the plant has been performed, including the new equipment. The proposed method is able to detect the risk associated with the introduction of a closed barrier, and results are coherent with analyses performed on the same plant with other models [24].

## 2. Materials and Methods 

**Scheme 1:**
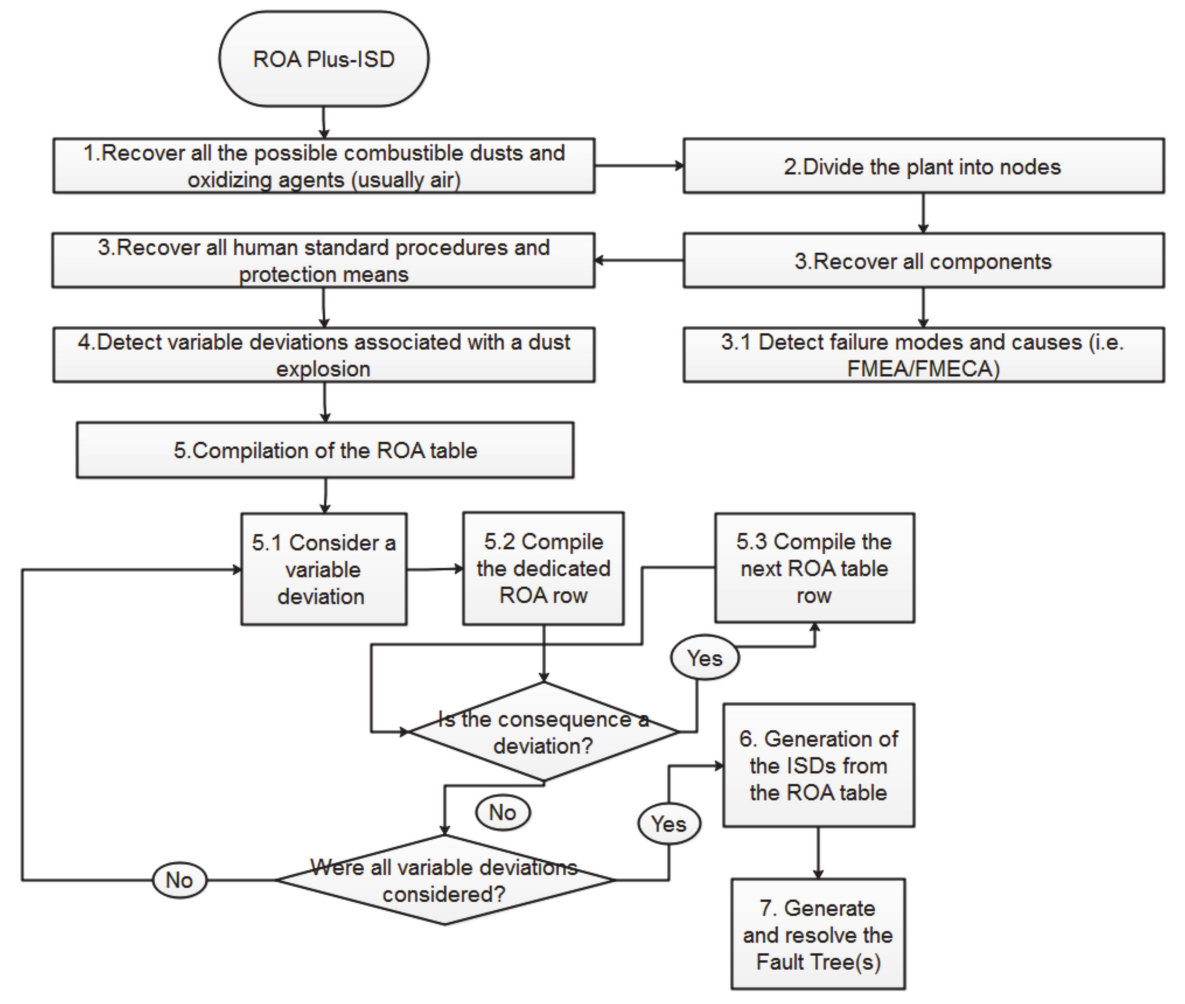
Flowchart of the ROA Plus–ISD method for dust explosions.

The flowchart of the ROA Plus–ISD is presented in Scheme 1. The procedure can be organized more concisely in the following steps:

- recovering of oxidizing and combustible agents
- classification of the plant in nodes
- collection of components and computation of the FMEA
- collection of human interaction with the system, with possible human errors associated
- identification of process variables and ignition sources for dust explosion
- compile the ROA Plus table
- generate ISDs and the associated FTs
- perform FTA

For what concerns the preliminary part of the method, technical details are then reported. The first step consists of listing all the substances present inside the plant, including combustible dusts and oxidizing agents. In this part, it is also crucial to recover all information about dust explosivity, therefore it is necessary to know Minimum Explosive Concentration (MEC), Minimum Ignition Energy (MIE), Limiting Oxygen Concentration (LOC) [25], Minimum Ignition Temperature (MIT) [21], deflagration index (K_St_) [26]. 

If certain dust concentrations and/or other characteristic values can be reached inside a plant (in any possible process), then performing a risk analysis concerning dust explosion is necessary. After this, the system should be divided into nodes, possibly distinguishing among different functions of the available equipment. Now, in order to define the basic events that will trigger potential accidents, all components (such as switches, safety systems, elevators, conveyor belts, reactors, valves…) must be retrieved and listed (it would be ideal to work with the Piping and Instrumentation Diagram, when available). From this list, failure modes and effects should be defined. In this sense, FMEA/FMECA [5] is a very good tool to accomplish this task, and it is highly suggested for correct implementation of the method. FMEA is performed by completing the scheme reported in Table 1. It is important to notice that failure causes will be the basic events that will appear in the final Fault Trees.

Besides, human-based procedures must also be recovered, since they can be crucial for safety issues [27], including both regular ones and protective actions (such as a manual loading or hand valves opening). Human procedures and errors can be treated in an FMEA-like module, indicating for each human interaction with the system the possible errors, which can be also recovered from literature databases [5,28].

The following step consists in defining the process variables involved with dust explosion. Table 2 summarizes these deviations, evinced from the well-known dust explosion pentagon.

**Figure 1:**
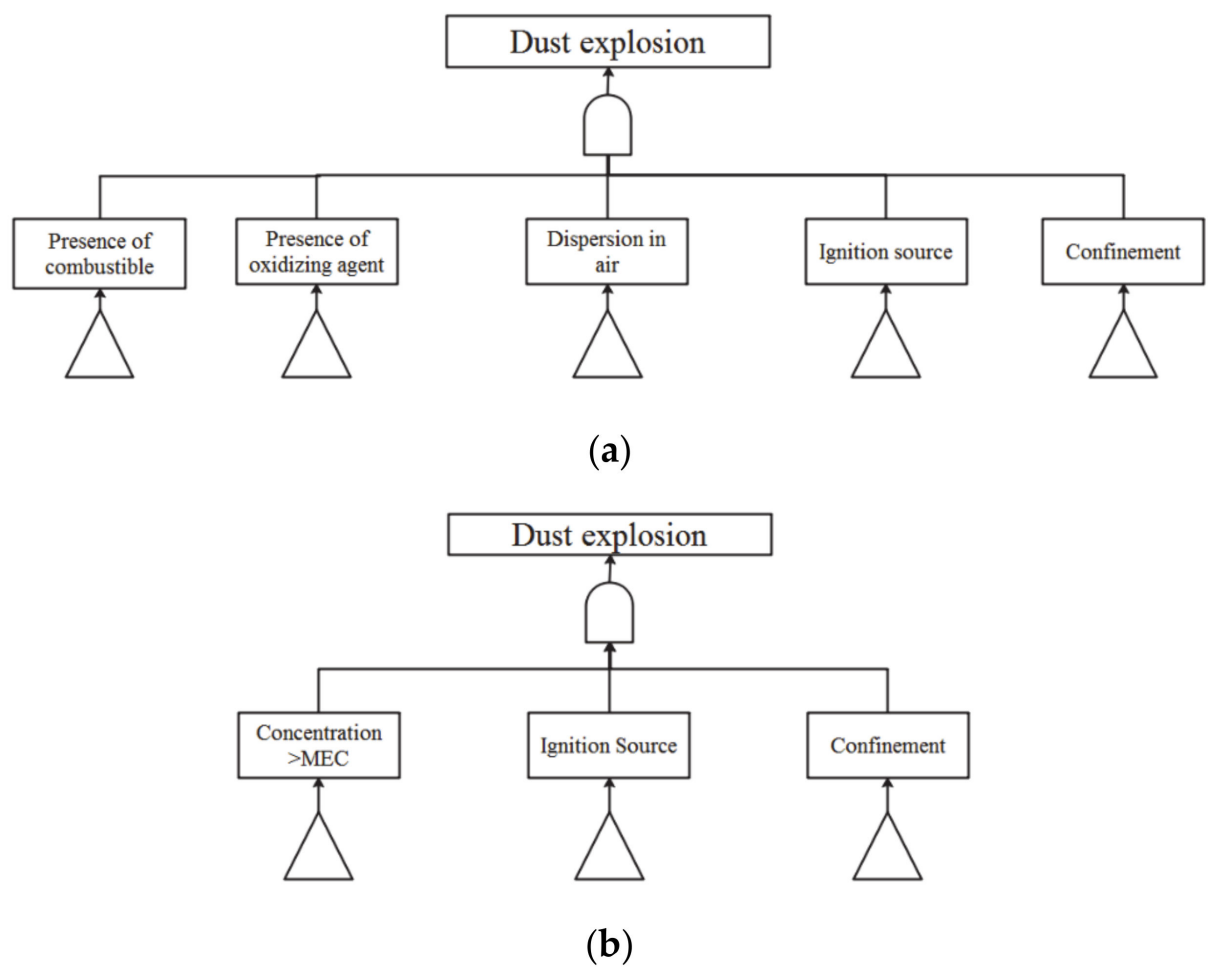
Dust explosion Fault Trees. (**a**) The basic structure based on the dust explosion pentagon; (**b**) The FT structure used for the ROA Plus–ISD.

It is important to note that these process variables should be also related to specific equipment and substances. The variables reported should be defined in a way so that they can be included in a ROA table so that ISDs can be generated. Figure 1a exhibits the most straightforward Fault Tree to define a dust explosion. However, this structure may have some implications with the application of the ROA Plus–ISD, since it is generally hard to define in terms of process variables a sufficient presence of combustible, oxidizing agent and dispersion. Thus, these three data can be summarized with the variable “concentration”. Moreover, a dust explosion can occur when the concentration of dust in the oxidizing agent reaches levels above a precise threshold limit value, which is the Minimum Explosive Concentration [19,21]. In this way, it is possible to represent through process variables the presence of dust inside the explosivity region. Other variables listed in Table 2, such as flow, mass and pressure, are variables that can cooperate to generate airborne dust, but since they are already process variables, the ROA method is already designed to face with these types of interactions. High masses and flow can lead to deposits, which can potentially generate dust clouds. Dispersion in air, can be due to several factors, such as primary explosions, equipment dedicated to dust conveyance and interaction with airflows [21], which are represented by high pressure or high flow. Confinement is usually related to the existence of enclosures in the plant, that is, low volume. While this is a process variable that is definitely not monitored, it is interesting to consider it in case of a new plant parts design. Also, a low available volume often appears as a Boolean variable, indicating whether or not confinement is present inside the facility. Regarding the definition of the ignition source, this issue is also complicated: sources of ignitions are multiple, such as electrical sparks, friction sparks, brush discharges, fires and hot surfaces, which can expose the dust cloud to values greater than the MIT [21]. Unfortunately, most of these are not easily represented by deviations of process variables.For this reason, it is more convenient to consider each ignition source as a cause which should be developed in the ROA table as a variable deviation, in a way that it can be then associated to other process variables or basic failures/events. Hence, all the possible ignition sources should be carefully listed a priori before starting the ROA table compilation.

Once all these preliminary tasks are accomplished, the core part of the method is the execution of the Recursive Operability Analysis (Plus), which consists of the computation of the table represented by the standard format, shown in Table 3. This table is substantially equal to the ROA-CCD table [17,18], which is a more complex version with respect to the classic ROA table [13,16,29]. It can be noticed that Column 1 (Node Deviation Variable), basically condenses the 3 elements of the classic HazOp scheme in a single one, including the plant node, the physical variable, and the deviation type. Column 2 represents the causes of the deviation, while Column 3 contains the consequences on the plant (considering the failure of all protective systems). The consequence can be either a modified process variable (such as high pressure following a high temperature) or a Top Event (such as a fire). The table accounts for the presence of eventual protective means, and it separates alarms, which generally require operator intervention and automatic protections. If consequences or causes appear as deviations of additional process variables, the analysis must be carried out until only basic events and Top Events are found.

**Scheme 2:**
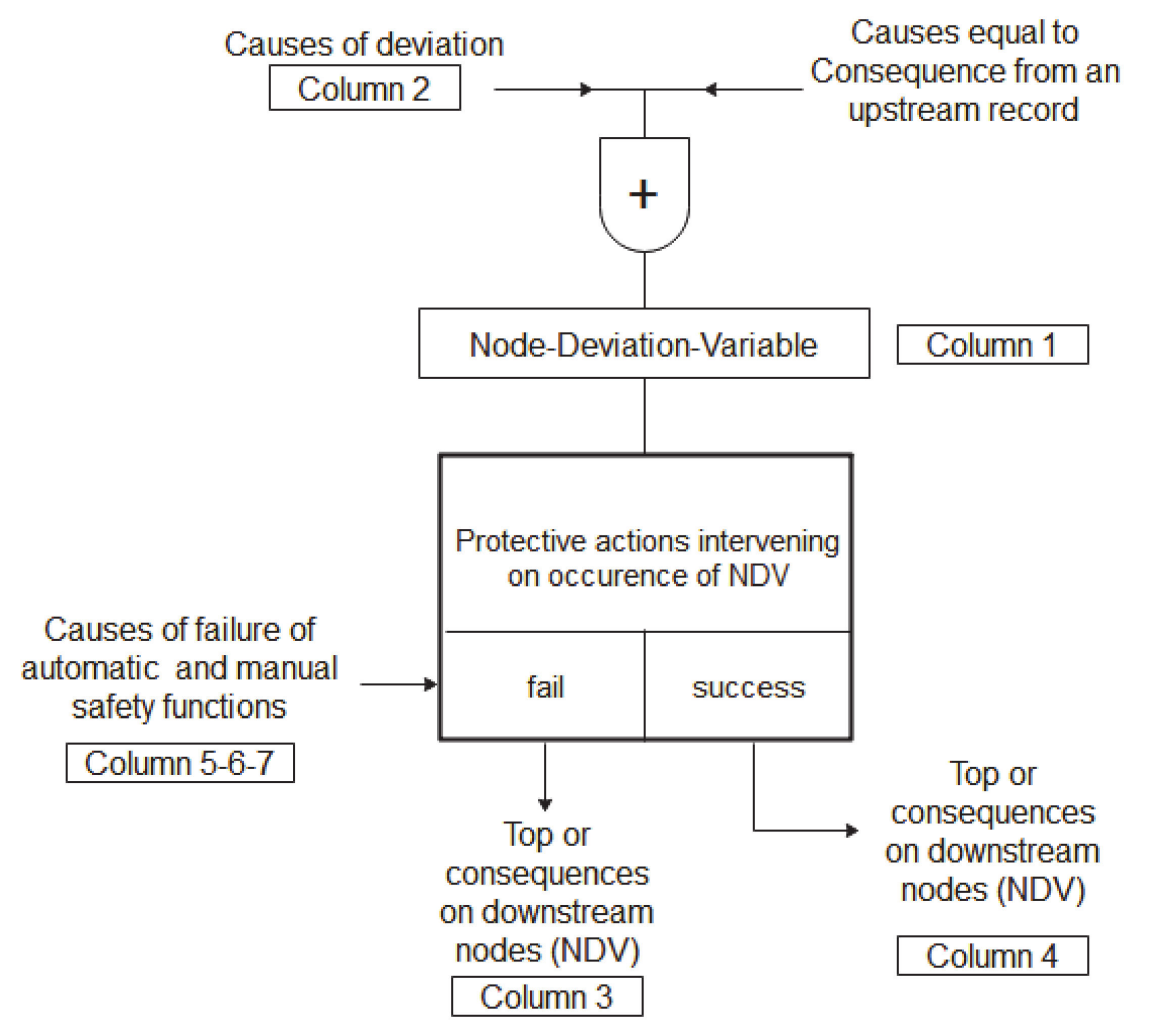
ISD generation from a ROA-CCD record. (NDV—Node Deviation Variable)

**Scheme 3:**
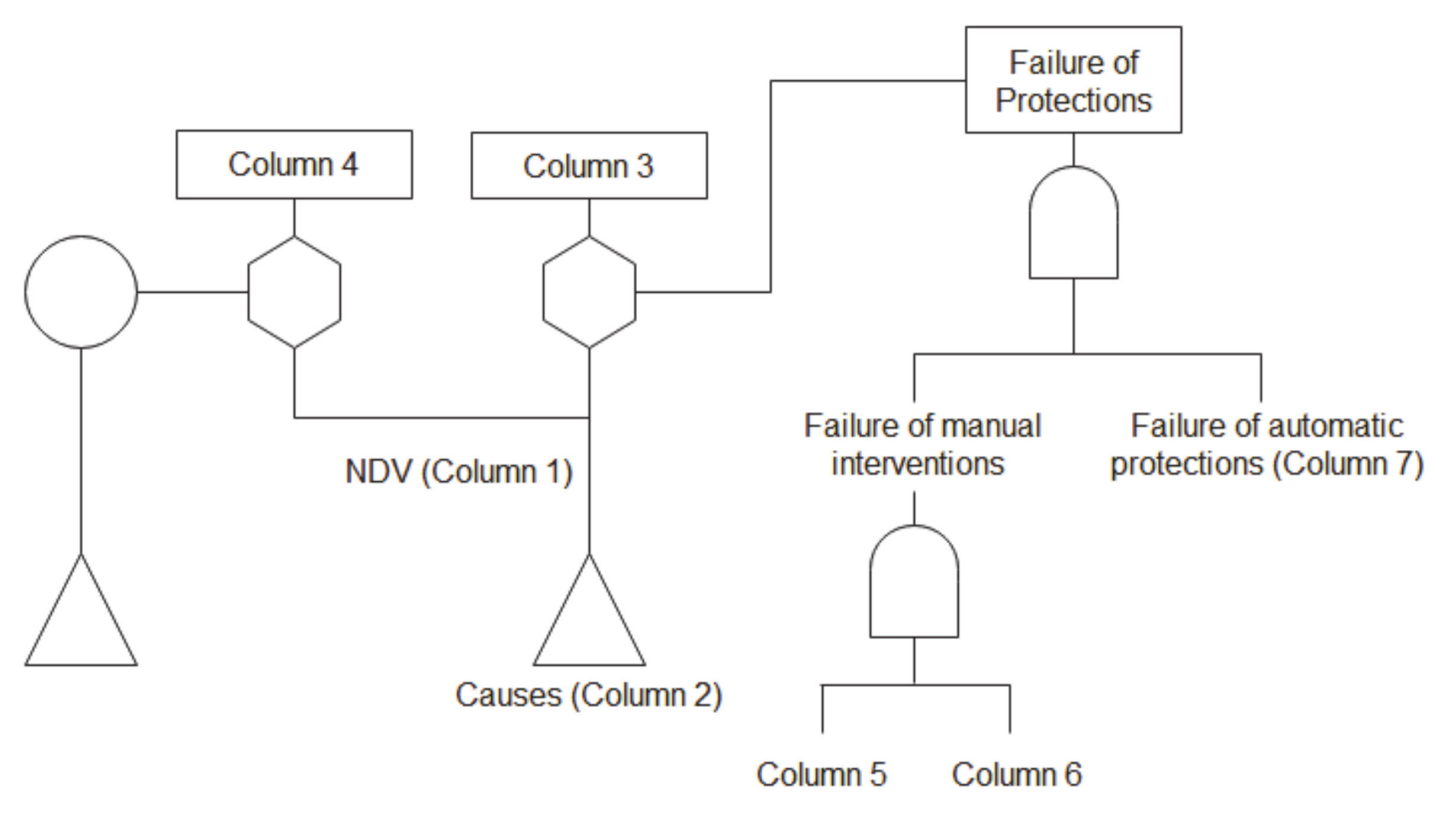
ISD generation from a ROA Plus–ISD record.

From this point, the main difference between ROA-CCD and this new model come out: according to the ROA-CCD method, after the ROA table is complete, from each record (that is, a row) of the table, a Cause-Consequence-Diagram can be generated, as shown in Scheme 2. It can be noticed that the CCD contains information about the plant state in case of protective means working correctly, allowing for a more proper analysis. However, CCDs can be difficult to be interpreted and connected to generate a Fault Tree. For this reason, it is easier to implement Incidental-Sequence-Diagrams, as shown in Scheme 3. 

The ISD contains the same information of a CCD, but it is already structured to generate a Fault Tree, allowing for faster generation of Fault Trees. The implementation of Column 4 inside the logic structure acts as a NOT gate: Column 3 represents the state of the plant according to the failure of protections, and column 4 is the state of the plant when protections work correctly (that is, the complementary part). Note that, for simplicity of reading, Column 4 can be eventually removed from the ISD, if the protective means have no impact on the state of the system state.

When all ISDs have been generated, they can be linked together, matching compatible causes and consequences, and a final fault tree hence comes out for each detected top event. For the solution of the fault trees, unavailability/failure rates of a component/human errors are required, which can be eventually retrieved from the literature. The final fault tree can be then solved and analyzed with dedicated software. In this work, OpenFTA 1.0 (Formal Software Construction Ltd., Cardiff, UK) was used. 

### 2.1. Case Study

The first explosion at the Imperial Sugar manufacturing facility (Port Wentworth, GA, USA) is considered as the case study of this work. On 7 February 2008 a sugar dust explosion occurred at the first floor of the packing building, exactly in the enclosed steel belt conveyor under the granulated sugar storage silos. Seconds later, another massive secondary dust explosion propagated through the entire building. The Imperial Sugar factory was a raw cane sugar refinery. The U.S. Chemical Safety and Hazard Investigation Board (CSB) investigated and reconstructed this event, the final report was considered for the recovery of the information about the process and the explosion. 

At the Imperial Sugar plant, a system of screw and belt conveyors, and bucket elevators transported granulated sugar from the refinery to three 32-meter-tall sugar storage silos. It was then transported through conveyors and bucket elevators to specialty sugar processing areas and granulated sugar packaging machines. Sugar products were packaged in four-story packing buildings that surrounded the silos or loaded into railcars and tanker trucks in the bulk sugar loading area [30].

**Figure 2:**
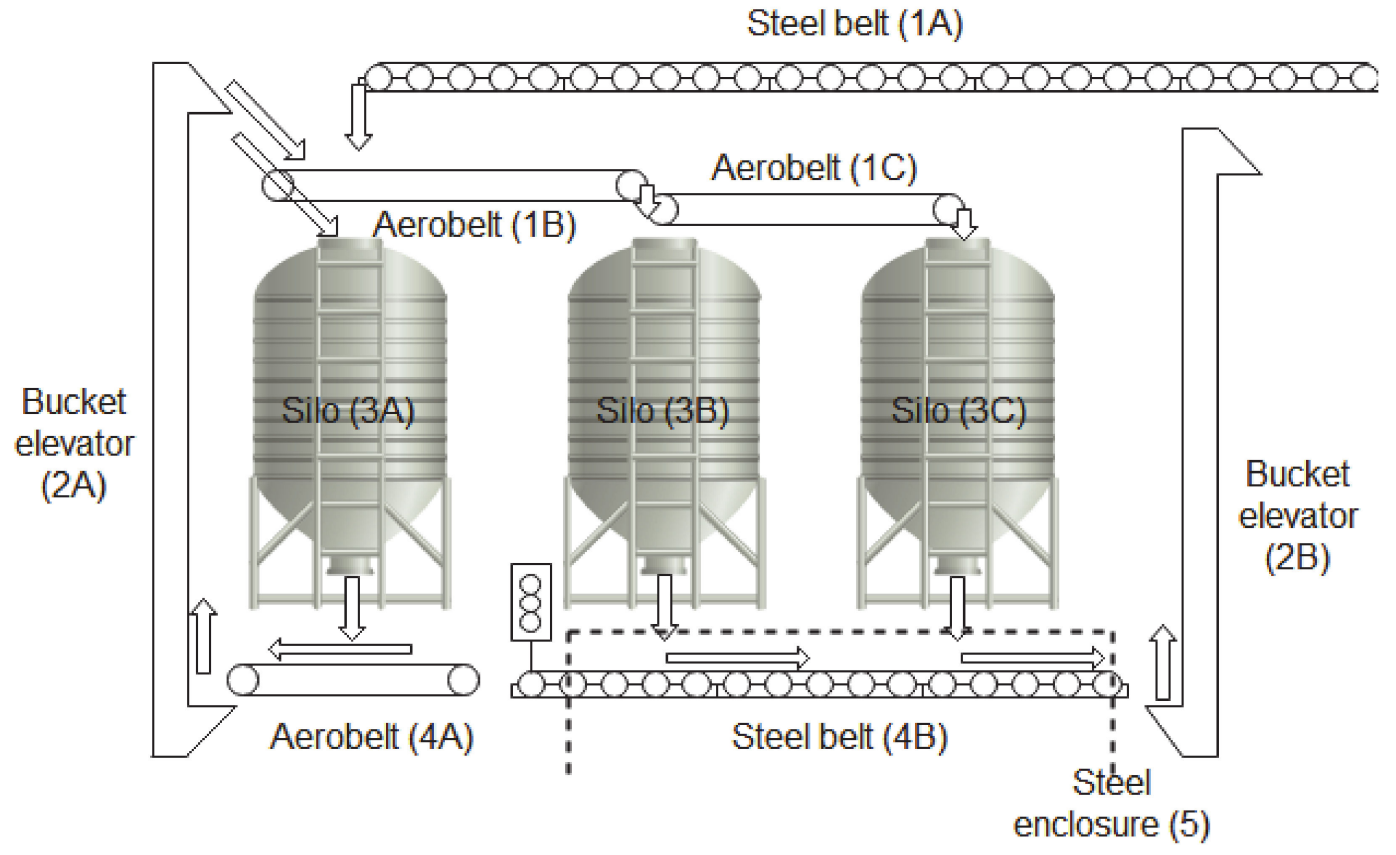
Granulated sugar supply and discharge through the silos. Arrows indicate where sugar flows. In brackets, the dedicated node is indicated.

As shown in Figure 2, on the first floor of the packing building there were three 12-meter diameter, 32-meter-tall concrete silos where the granular sugar produced in the refinery building was conditioned and stored. Under the silo floor, a belt conveyors tunnel which contains one Aerobelt^®^ conveyor (which discharged the sugar into the west bucket elevator pit), and a steel belt conveyor (which transported granulated sugar from silo 3B and 3C to the packaging production bucket elevator pit located at the east side of the silo 3C) were located. To avoid possible contaminations of the sugar, in 2007 Imperial sugar decided to enclose the belt conveyor under silo 3B and 3C with stainless-steel panels. Less than one year later a dust explosion occurred into the enclosed belt conveyor.

The surveys carried out by CSB investigators showed that, in addition to the lack of maintenance and housekeeping on the plant, the conveying granulated sugar on the steel belt conveyor generated airborne sugar dust. Also, investigators have discovered, interviewing workers, that sugar lumps often clogged the silo outlet pipe and blocked the movement of the sugar on the belt. As a consequence, the sugar spilling off the belt was released as airborne dust.

The enclosure was not equipped with deflagration vents to reduce safely the overpressure if airborne sugar dust was ignited. Moreover, no dust removal system to prevent the concentration of airborne sugar being exceeded the MEC inside the enclosure was installed to avoid an explosion.

The CSB investigators learned that three days before the explosion, workers were cleaning sugar lumps lodged in the silo 3C. During the cleaning activities, sugar continued to flow on the steel belt conveyor from silo 3B upstream of the silo 3C discharge chutes. The sugar lumps created a sort of dam on the conveyor that caused a sugar spill off the belt. As a result, this leak caused an accumulation of spilled sugar inside the covered conveyor and part of the steel belt was covered by the dust. Inside the tunnel sugar dust accumulated above the MEC.

Interviewing the operators, the CSB investigators found that bearing on the steel roller supports sometimes malfunctioned and warmed up, they deduced that the possible source of ignition was a hot bearing inside the enclosed conveyor. The high rise in the temperature of mechanical parts can actually trigger airborne sugar dust, especially if the dust remained in contact with the hot surface for many seconds, as it likely occurs in an unventilated enclosure. Also, if sugar in contact with a hot surface begins to smolder, combustion gases that are released will mix with the airborne sugar dust and decrease the ignition temperature below the ignition temperature of pure sugar dust [21].

### 2.2. Application of the ROA Plus–ISD method

The procedure is applied here step by step.

#### 2.2.1. Detection of Combustible and Oxidizing Agents

Sugar dust is the only combustible material present in the facility, oxygen (air) is the oxidizing agent. Explosive properties of sugar dust are reported in Table 4. All the reported values can be reached inside the facility: the MEC can be reached due to lifted dust deposits, air compressor, human-based procedure. The MIT is around 400 °C: if a sugar deposit catches fire, this temperature can be easily reached. The MIE is fairly high, but that short circuits, electric and friction sparks can release such energies.

#### 2.2.2. Identification of Nodes

The whole room represented in Figure 2 is the plant section dedicated to sugar dust collection and conveyance. The analysis will include the steel panel used to protect the lower sugar line. The system is divided into five nodes: Node 1A–1B–1C (upper conveyor belts), Node 2A–2B (elevators), Node 3A–3B–3C (silos) and Node 4A–4B–4C (lower conveyor belts), Node 5 (steel enclosure). In particular, among the conveyors both classic roller conveyors and Aerobelt^®^ conveyors are present (1B–1C and 4A). Aerobelt^®^ conveyors exploit air cushions in order to move a powdered material, and they are designed to minimize leaks and dispersions. 

#### 2.2.3. Recovery of Failure Modes and Frequencies from a FMEA

Components present inside this part of the plant are: switches (Node 4), steel panels (Nodes 1, 3, 5), conveyor belts (Nodes 1–4), rollers (Nodes 4A, 4B, 4C), fans (for Aerobelt^®^ conveyors 4A, 1B, 1C), discharge valves (Nodes 3A, 3B, 3C). In addition, human operators can access and interact with Nodes 3–4–5. Table 5 reports the FMEA analysis for the involved components. Failure rates were recovered from literature, when available. According to the CSB report [30], some events may be considered unlikely (such as an electric spark from the hand switch. In this case, for the simulation, a conservative value of 1 × 10^−6^ h^−1^ was considered. It is known, from plant information, that sugar clogging would occur at the outlet of silos 3B and 3C, stopping the sugar conveyance. Whenever this occurred, operators should stop the belt with a dedicated switch, and manually clear the way out with a steel rod. This operation was referred to as “rodding” [30]. In the regular handling of the process, this is the only human intervention foreseen.

While it is interesting to analyze the impact of this operation on the process (for example, operators may trigger a friction spark with a misuse of the steel rod, the model accounts only for regular protection systems. In this case, possible human errors are listed in Table 6.

#### 2.2.4. Indication of the Most Probable Ignition Sources

Considering the plant, the following have been listed as the most probable ignition sources: electric sparks, friction spark, hot surfaces or a local fire. Electric sparks can be generated by a faulty switch, which is present around the bottom belts line. Friction sparks can be generated by operators, through a bad execution of the “rodding” procedure, or by falling metallic parts. This issue is more critical in the bucket elevators, where buckets can get loose and fall upon other metallic parts. Hot surfaces and fire can be generated through friction between sugar and roller bearings, or by worn roller bearings, if dust deposits are available. This occurs wheter the dust deposit, which acts as a layer, reaches the layer ignition temperature (LIT). Unfortuantely, specific tests to investigate the LIT of the sugar processed in the facility were never performed. According to a Chemwatch Safety Data Sheet for icing sugar, the LIT can be considered to be about 450 °C [32]. 

#### 2.2.5. Process Variables

All relevant process variables are listed in Table 7. Inside the plant, sugar flows are present in silos 3A–3B–3C, where sugar is sent from silos to the dedicated belts through discharge valves. Sugar flows are also present on buckets elevators (2A–2B), and on the conveyor belts (1A–4A–4B). Sugar mass indicates deposits, which can occur mainly inside the conveyor belts 4A–4B, where it can slip through the bearings. 

**Figure 3:**
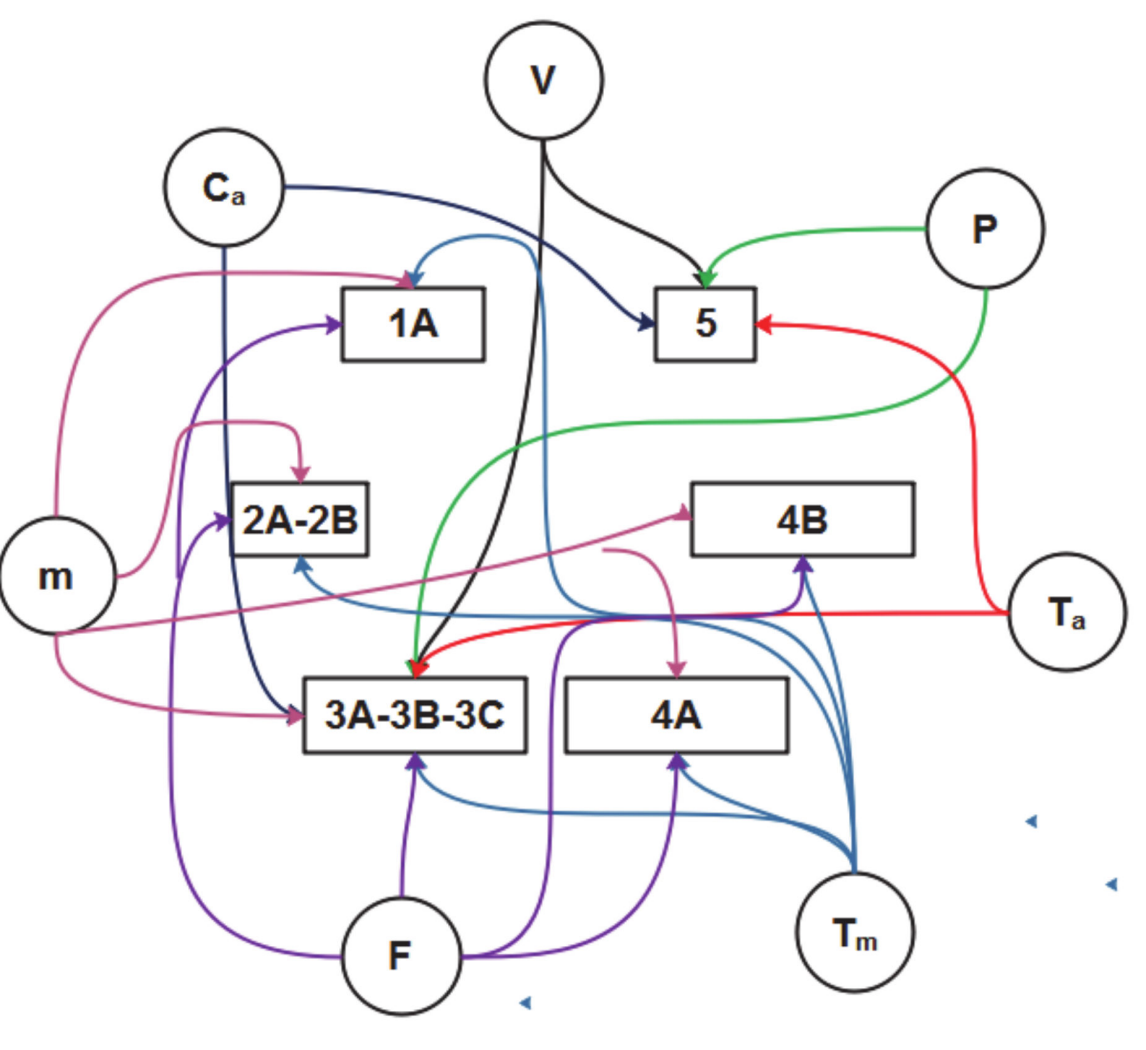
Couplings among process variables (circles), and process nodes (boxes). *F*—sugar flow; m—sugar mass; *C_a_*—airborne sugar dust concentration; *V*—volume available; *P*—pressure; *T_a_*—air temperature; *T_m_*—temperature of mechanical parts.

Airborn sugar concentration, and air temperature are related to silos 3A–3B–3C, and the enclosure (Node 5). In this case, Nodes 4A–4B are very close to Node 5, since the belts are contained in the enclosure. Pressure is also associated to the dust cloud, which can be present inside all the silos and in the enclosure. The temperature of mechanical parts is referred to generic equipment components, which can act as a hot wall for dust ignition. Volume is strictly referred to confinement, and it is present inside the silos and in the enclosure. Connections among proves variables and nodes are graphically represented in Figure 3.

## 3. Results and Discussion

ROA Plus–ISD has been applied to the facility involved. In this section, results concerning the steel conveyor belt (Node 4B) and the enclosure (Node 5) are reported. These nodes represent the major criticalities as they represent the zone that witnessed the installation of the steel enclosure, and it is where the primary explosion took place [30].

The ROA table is reported in the Appendix A: Table A1 is the ROA associated with the steel belt (Node 4B), Table A2 represents the analysis on the enclosure (Node 5). In order to make an easier reading of the table, unimportant deviations or redundant elements were omitted. For example, the deviation “low temperature of mechanical parts” is extremely unlikely, and it eventually brings to trivial consequences. Therefore, all deviations experiencing this issue were not then reported.

According to the ROA table, the main deviations associated with a potential sugar dust explosion can be clearly seen: high concentration, air temperature, pressure, confinement, and ignition sources. In this case, sugar dust can become airborne over the regular handling of the process, due to both conveyor belts and sugar flow from the discharge duct from the silos. For this reason, a high concentration of sugar is a direct consequence of having dust deposits. Additional factors that can lift sugar dust in the air are human operations, such as cleaning operations with compressed air, or the “rodding” operation, where an operator is supposed to clear the outlet of sugar after a clogging event. Cleaning operations with compressed air are not normally performed in this part of the plant and the effect of a protection measure (that, is the “rodding” operation after a clogging of the outlet line) is seen in the ROA table.

About the process variables involved, high airborne sugar concentration *hC_a_* indicates a concentration above the MEC (Table 4). A high temperature of mechanical parts *hT_m_* indicates temperature below LIT but higher than normal temperature, where very high temperature *hhT_m_* means temperatures above the LIT, allowing for sugar deposits ignition. Low volume indicates the presence of confinement, which allows for both increasing airborne sugar concentration and pressure accumulation. In this case, confinement is given by the presence of the enclosure. If the enclosure was not present, higher concentrations would be reached only locally, which is inside the silos and in the proximity of sugar deposits and discharge points. While an explosion inside a silo is indeed a critical issue (but it is an object of the analysis dedicated to the specific silo node), for what concerns the 4B Node the absence of the enclosure would mean that only local fires are a potential accident, given the lack of confinement.

As recalled previously, the most important ignition sources were identified as high temperature from a sugar fire, intended as temperature > MIT (*hhT_a_*), electric and friction spark. High temperatures are well represented by regular deviations, while electric and friction spark may not be necessarily basic events, and they must be developed separately. Considering the equipment, an electric spark can be generated by the faulty hand switch, which is highlighted in the FMEA (Table 5). This event was reported as unlikely [30]. For what concerns the overpressure generated, according to literature data, sugar dust exhibits deflagration indexes that can range from 30 to 150 bar/m/s [30,33], depending upon the moisture content and particle size distribution. This means that sugar dust explosions can lead to severe damage to both equipment and people. For this reason, it is considered as a level of concern for high pressure, a value equal to 2.76 kPa, corresponding to a potential enclosure collapse [31].

From the analysis, four top events have been identified: conveyance of sugar blocked (TE1), sugar spillage (TE2), local fire (TE3), primary explosion (TE4), dust cloud formation (TE5). The blocking of sugar conveyance is due to the sugar clogging, which appears to be a pretty frequent event within the process. In this case, a protective measure is foreseen, that is the “rodding” operations, which require operators to stop the belt motion, and manually clear the sugar outlet. A local fire, on the other hand, can be generated through dust deposits in combination with any ignition source. In particular, dust deposits that fall inside the bearing of the rollers can contribute to dust ignition, compromising their friction. A dust explosion, instead, requires confinement and high dust concentration in combination with a triggering event, which is a local fire or a spark. Also, sugar clogging can determine a visible sugar dust cloud, which is by itself a critical issue that requires attention. From this ROA table, ISDs can be automatically generated.

### 3.1. Fault Trees from ISDs

**Figure 4:**
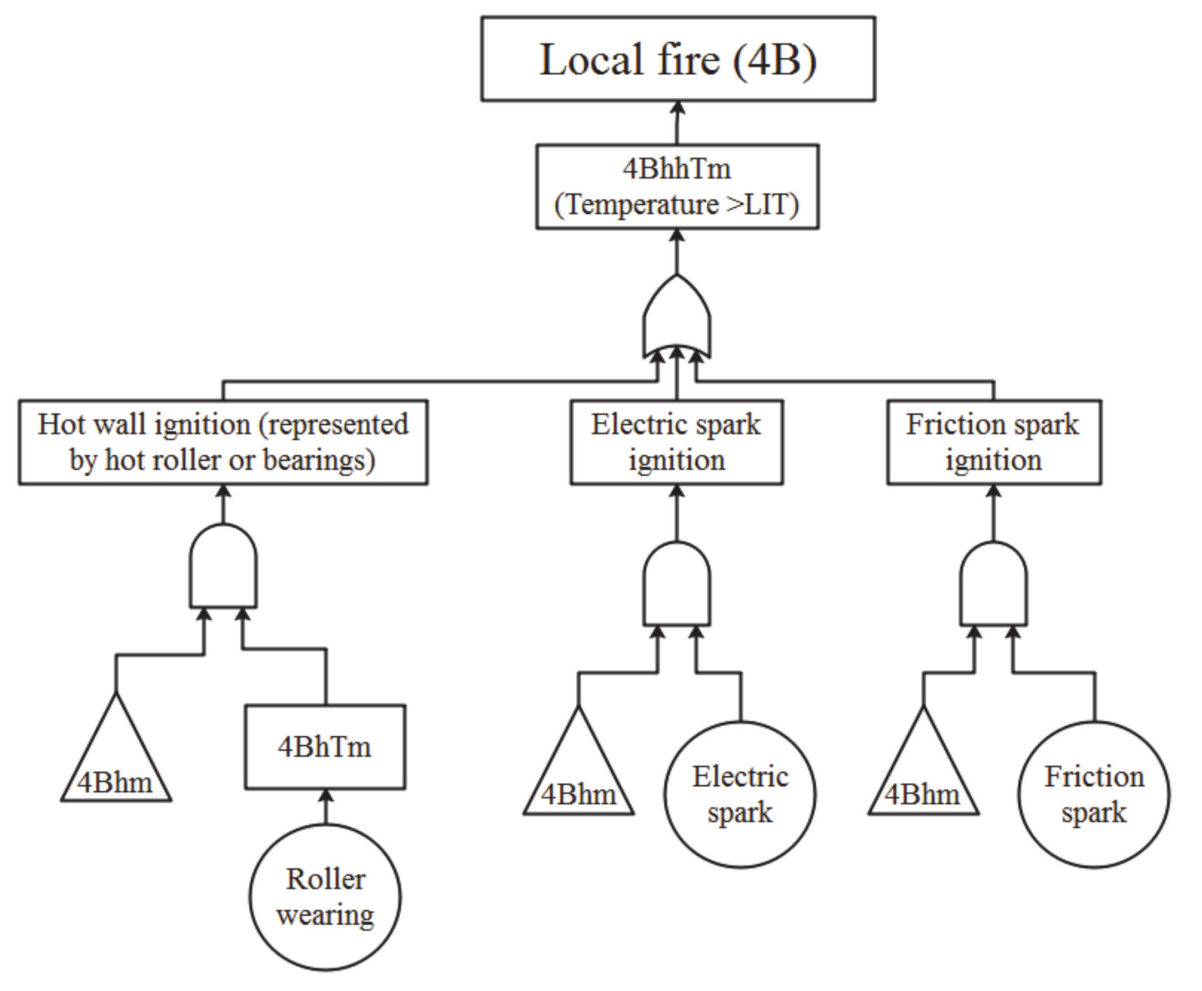
Fault tree for the local fire top event. 4Bhm—high mass inside node 4B; 4BhTm—high temperature of mechanical parts inside node 4B.

**Figure 5:**
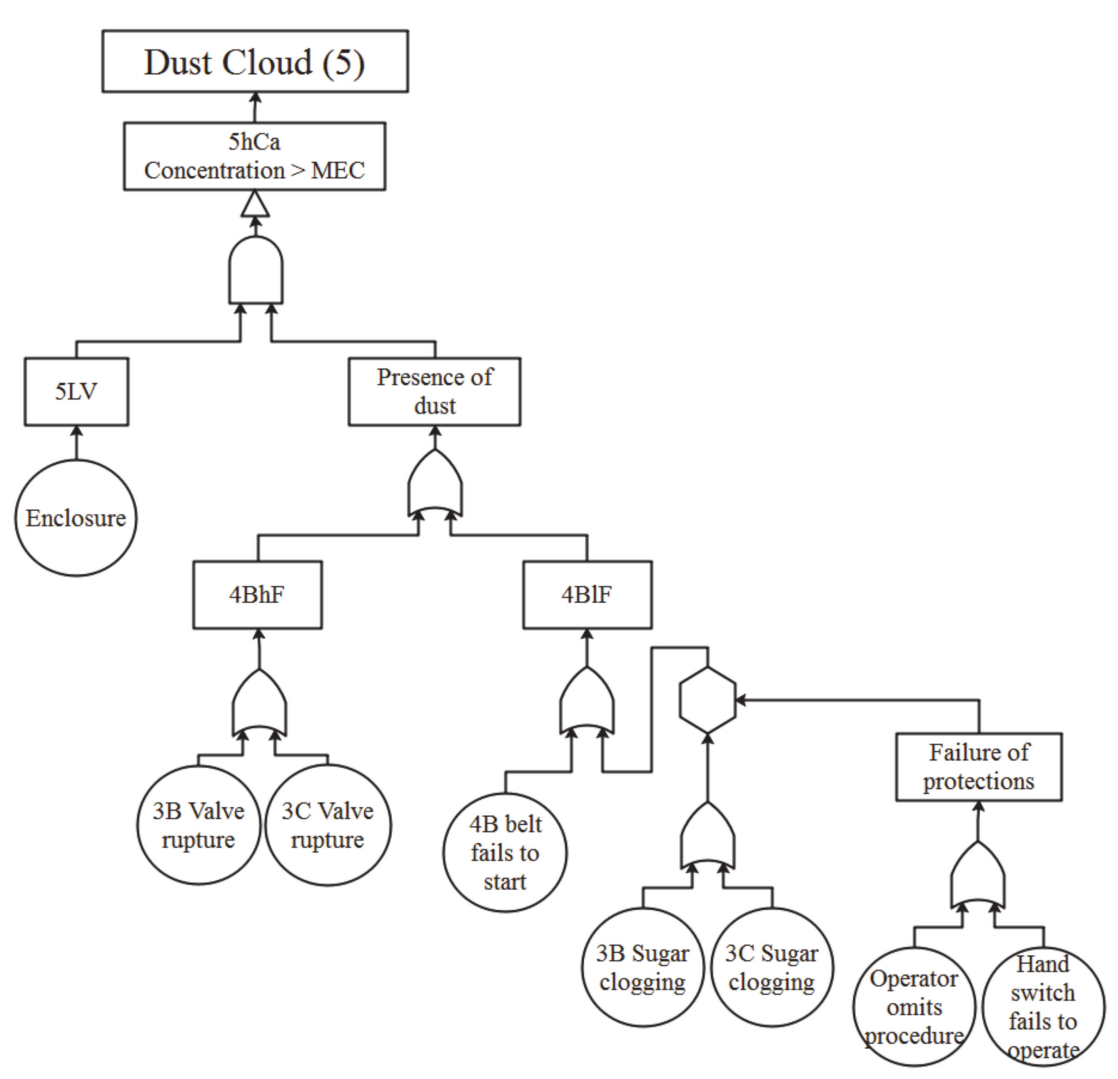
Fault tree for the dust cloud formation top event. 5LV—low volume inside node 5; 4BhF—high sugar flow inside node 4B; 4BlF—low sugar flow inside node 4B.

**Figure 6:**
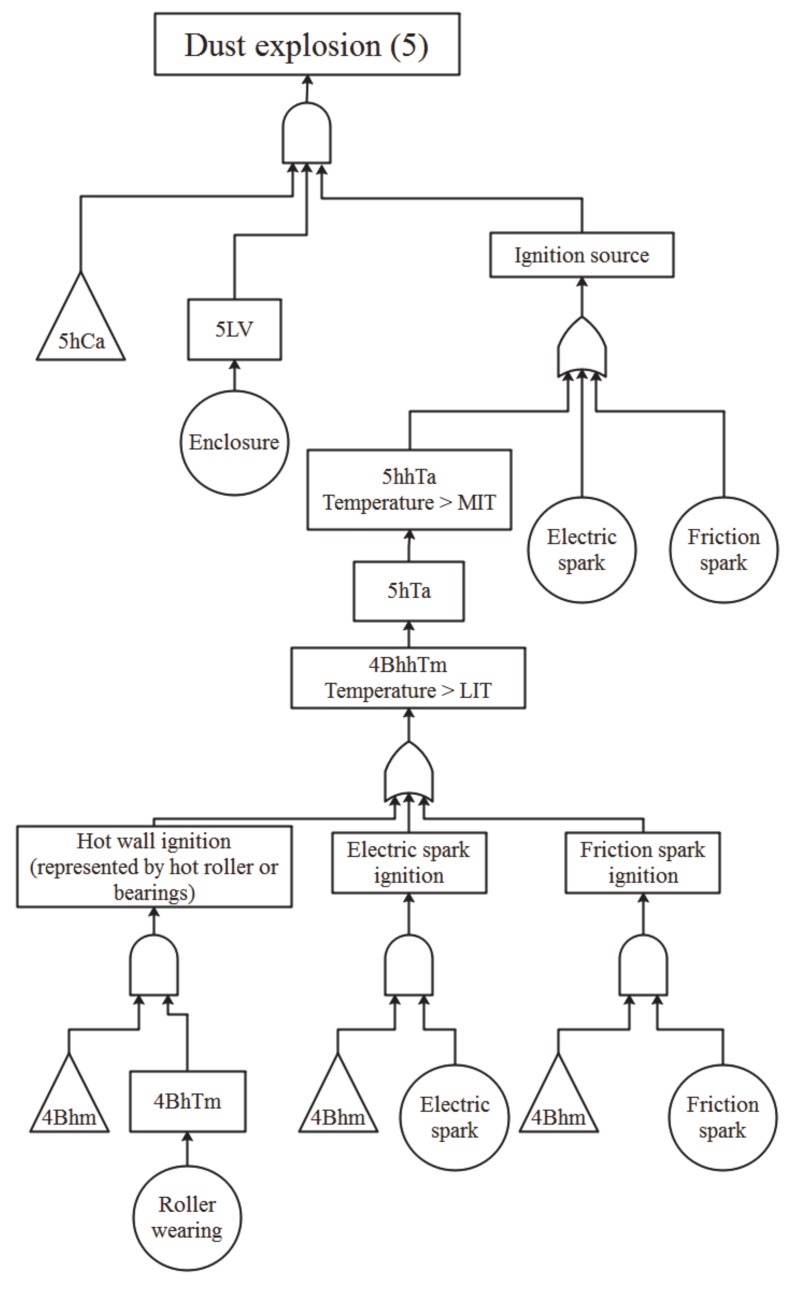
Fault tree for the dust explosion top event. 5hCa—high concentration inside node 5; 5hhTa— very high air temperature inside node 5; 4BhhTm—very high temperature of mechanical parts inside node 4B.

**Figure 7:**
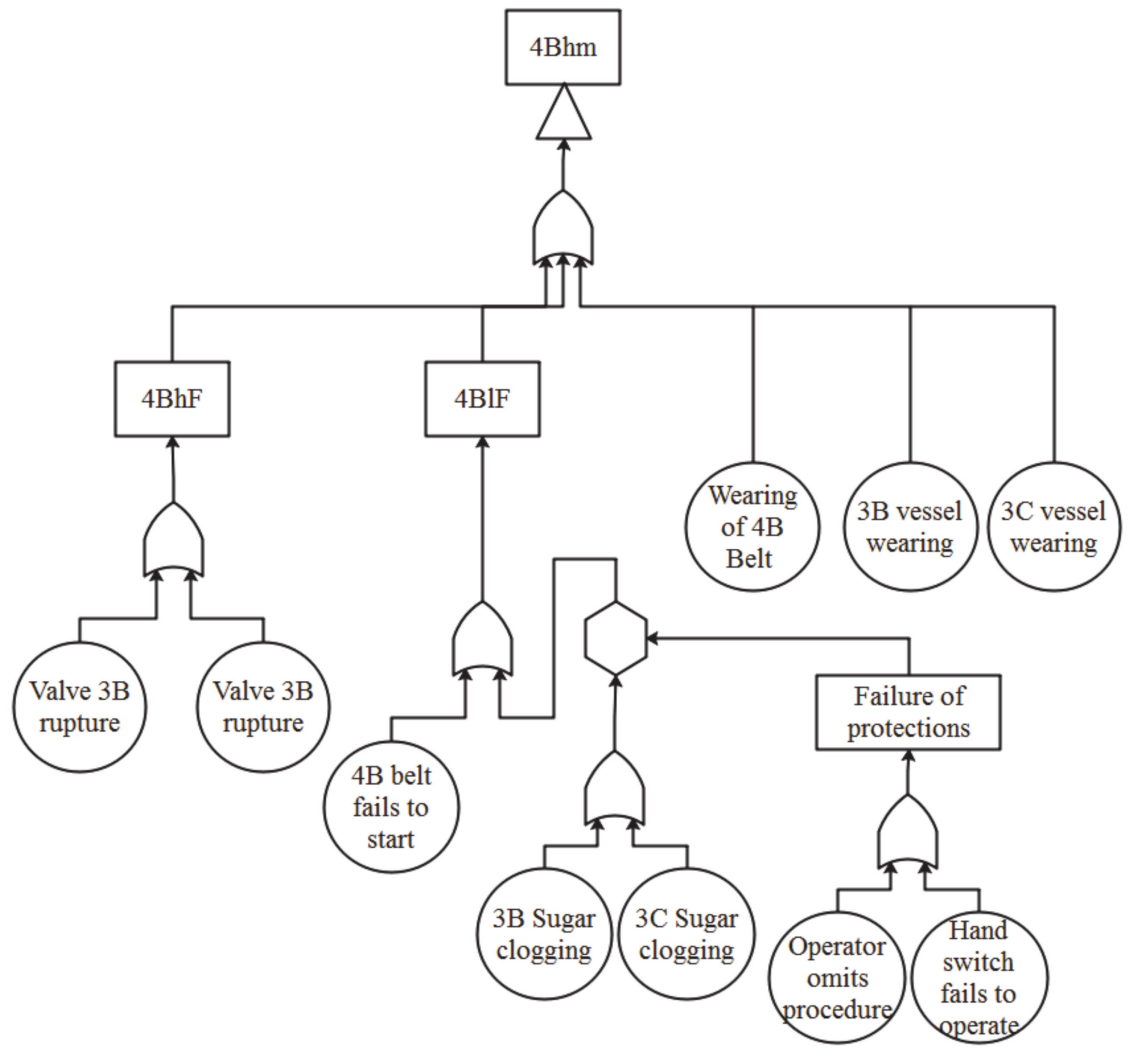
Transfer gate for the 4Bhm deviation.

ISDs can be generated for the top events found. For safety concerns, the most crucial top events are local fire (TE3), formation of a dust cloud (TE4), and the occurrence of a primary dust explosion (TE5). Figures report the complete fault trees, generated by linking all the related ISDs: Figure 4 shows the FT for local fire, Figure 5 shows the FT for dust cloud formation and Figure 6 the FT for a primary dust explosion. For clarity, the event 4Bhm is shown separately with a transfer gate in Figure 7. These FTs can be now be analyzed with dedicated software, such as OpenFTA.

Significant process variables, such as concentration and various temperatures have been associated with the critical values they are referred to. It can be noticed in Figure 5 and Figure 7 that the ISDs generated do not include Column 4 of the relative ROA table, which indicates the presence of protections. This part was not included in the graphical representation for simplicity’s sake, because when protective actions work correctly (operators performs the standard “rodding” procedure), system goes back to its normal functionality, without severe consequences for the plant state.

### 3.2. Fault Tree Analysis

Fault trees generated have been implemented and analyzed with OpenFTA 1.0. This software performs an FTA and gives as results minimal cut sets, probabilities, and the importance of single basic events. However, in order to have a proper estimation, mission time and unreliability functions must be defined. A mission time of 1 year was considered, this is a reasonable value considering the introduction of a new element in the process (the steel enclosure). Under these circumstances, it is reasonable to consider as negligible, maintenance and repair. Hence, unavailability *P* of an *i-th* component can be estimated through a Poisson distribution [34], defined in Equation (1):

(1) $$P_{i}\left(t\right){=1\;-\;e}^{-\lambda t}$$

where *t* is the mission time and *λ* is the failure rate of the component. For human errors, direct probabilities are given from the database. All probabilities for the components listed in the FMEA (Table 5) are shown in Table 8. Short IDs for each failure are also indicated: the first letters indicate the node, and the others the type of basic event. As an example, 5En stands for “enclosure in Node 5”. Events considered unlikely have been treated as elements with failure rate equal to 1 × 10^−6^ h^−1^, resulting in a probability over one year equal to 8.72 × 10^−3^ . The presence of the enclosure acts as a Boolean variable. After its implementation, the associated probability becomes equal to 1.

For the dust cloud event, eight primary events are involved, as shown in Figure 3. Seven minimal cut sets have been identified. Three of the Minimal Cut Sets (MCS) are of second-order, and the others of the third order. For the local fire event, 13 primary events are involved, with 30 minimal cut sets identified. Eighteen of these MCS are of second-order, and the other 12 are third-order MCS. For what concerns dust explosion analysis, 14 primary events are involved, with 21 minimal cut sets identified. Nine of these are MCS of the third-order and the other 12 of fourth-order. All results are summarized in Table 9.

As a general overview, it is well-known that high order MCS and a low number of MCS indicate an intrinsically safe procedure [35]. In this case, a high number of MCS is associated with local fire and dust explosion top events. However, this is highly due to the ROA Plus−ISDs, since it forces the analysis in considering all the possible ignition sources. MCSs are of low order (from 2 to 4), indicating a lack of protective measures. Nevertheless, the most severe event, which is the dust explosion, has the highest MCS orders.

The final estimated probabilities of the top events are the following: 1.13 × 10^−1^ for dust cloud formation, 2.75 × 10^−^^2^ for local fire and 6.73 × 10^−^^3^ for dust explosion. This means that a dust cloud is expected to be seen on average every 8.8 years, a local fire every 36 years, and a dust explosion every 148 years.

These results are consistent: it is reasonable to have an order of magnitude between dust cloud formation and local fire, and between local fire and dust explosion. According to such results, the generation of a dust cloud appears to be a pretty frequent event. This finds evidence in the accident reconstruction, as sugar was spread in the whole facility [30]. The most important factors that contribute to the formation of a dust cloud are the presence of the enclosure (100% importance, it is present in each MCS), and the hand switch failure in case of sugar clogging (63.19% importance). The least impactful events are the omission of procedure from the operator (3.53% importance), and the failure to start of the 4B belt (7.70% importance). For what concerns a local fire accident, the most important event is the wearing of the bearing of the 4B belt (94.27% importance). This fact is extremely interesting since it finds significant evidence in the accident analysis: operators reported that bearings would heat very often over their process phase [30]. The least impactful primary events are still the omission of procedure from the operator (0.88% importance), and the failure to start of the 4B belt (1.91% importance). For dust explosion, the presence of the enclosure has still 100% importance, since it is the only cause of confinement inside this node. Bearings wearing is still the most probable ignition source (99.24% importance). Such values are unacceptable in accordance with the ALARP principle [36]. These results have been compared with another risk assessment, performed on the same plant by Abuswer et al. [24]. In their work, they estimated a probability of occurrence of a dust explosion including a correction factor to estimated probabilities, based on the consequences of an eventual explosion. According to their simulation, the probability changed from 3.76 × 10^−2^ to 4.4 × 10^−5^ [24], after the application of the corrective factor. The result of this work brings to an intermediate value, being equal to 6.73 × 10^−3^, and it is very close to the result obtained by not including the QRMF correction. This work does not discuss the estimation of the magnitude of the events, nevertheless, events such as fires and explosions generate individual risk indexes which are different from the probabilities alone. In this sense, local fires and dust explosions with probabilities of occurrence equal to 2.75 × 10^−2^ and 6.73 × 10^−3^ over one year, respectively, will not generate individual risk levels lower than 1.0 × 10^−3^, which is the lower bound for the ALARP region. This analysis highlights the importance of considering the effect of plant changes, such as the addition of an enclosure on conveyor belts, by performing a proper risk assessment.

## 4. Conclusions

In this work, a method to generate fault trees specifically tailored for dust explosions has been developed and applied. The method consists of applying an enhanced version of the recursive operability analysis, called ROA Plus−ISD, as described in this paper. The aim of the work is to propose a highly structured procedure which can be implemented by a wide range of users, even without a deep knowledge of the topic. The method is based upon a structured information recovery in the preliminary part. Required information cover both technical characteristics, such as K_St_ and MIE, and plant information, as components and human-based procedures. These should be analyzed with a tool such as a FMEA, in order to define failure modes and causes, which will be the basic events of the final fault tree. After this preliminary work, the ROA Plus−ISD table can be completed, by analyzing each deviation formerly identified. Once the ROA Plus table is compiled, ISDs and FTs can be automatically generated. The whole procedure is almost automatic but analyst skills are always necessary for the generation of the ROA table. Nevertheless, some limitations of the model can be pointed out: while the model proposed is an attempt of making a highly automatized risk assessment tool, the method is very sensitive with respect to the preliminary part, which still requires a good knowledge of the process plant and physical phenomena involved. Especially in complex plants, all components must be carefully recovered and listed, or results would be otherwise unreliable. Also, ignition sources and human interactions may be complicated to be identified, and they should be thoroughly investigated and discussed. 

The procedure was used to perform the risk assessment of the Imperial Sugar Plant, focussing on the sugar conveyor line, a plant zone that witnessed a severe accident after the installation of a steel enclosure. From the results, it is highlighted the strong effect of the presence of an enclosure on the occurrence of serious accidents. Numerical probabilities and comments are in accordance with the reconstruction, providing unacceptable risk for each safety issue noticed. This suggests that if a simple but detailed analysis (such as a ROA Plus–ISD) of the risk associated to the plant had been carried out before making operative the plant itself, the catastrophic combustible dust explosion occurred at Imperial Sugar could have been avoided. 

## Figures and Tables

**Table 1:** Classic FMEA scheme.

**Component**	**Description of the Function**	**Failure Modes**	**Failure Causes**	**Consequences**	**Protections**		**Frequencies**
					**Alarms (Optical/Acoustic)**	**Automatic Protective Means**	

**Table 2:** Process variables related to the explosion pentagon. (x indicates a generic node, i indicates the equipment or substance which the variable is referred to; MEC—Minimum Explosive Concentration)

Condition	Variable Deviation/s	Symbol
Presence of combustible dust	High concentration (>MEC) High mass High flow	xhC_i_ xhm_i_ xhF_i_
Presence of oxidizing agent	High concentration (>MEC) High mass High flow	xhC_i_ xhm_i_ xhF_i_
Ignition source	High temperature Other sources	xhT_i_ -
Dispersion	High concentration (>MEC) High temperature High pressure	xhC_i_ xhT_i_ xhP_i_
Confinement	Low Volume	xlV_i_

**Table 3:** Classic ROA-CCD scheme. (Rec—Record; NDV—Node Deviation Variable; TE—Top Event)

Rec	NDV	Causes	Consequences Due to Protections Failure	Plant state with Protections Working Correctly	Protections			Notes	TE
					Manual		Automatic Safety Systems Actions		
					Alarm (Optical/ Acoustic)	Operator Actions on Components			
	1	2	3	4	5	6	7		

**Table 4:** Explosive properties of samples of granulated sugar collected from the Port Wentworth facility [30].

Property	Value
Minimum Explosive Concentration—MEC	115 g/m^3^
Minimum Ignition Temperature—MIT	360 °C–420 °C
Minimum Ignition Energy—MIE	>1000 mJ

**Table 5:** FMEA per the involved components and human errors. Frequencies are represented as failure rates. (h—hour; d—day; w—week; y—year)

Component	Description of the Function	Failure Modes	Failure Causes	Consequences	Protections	Frequencies	Remarks
4B Hand Switch	Stop of the roller belt	Load arc	Electric circuit wearing	The load arc can trigger dust deposits ignition		Unlikely [30]	
		Fails to perform	Switch wearing	The roller cannot be stopped for maintenance or emergency purposes		4 × 10^−6^ h^−1^ [5]	
3A–3B–3C (Silos vessel)	Sugar silo	Release of sugar	Vessel wearing	Sugar released on the ground		30 × 10^−6^ h^−1^ [31]	
		Flow blocked	Sugar clogging	Sugar flow is blocked by the clog, requiring human operator intervention	Operator intervention (“rodding”)	3 w^−1^ [30]	
3A–3B–3C (Discharge valves)	Sugar discharge	Fails to open	Fails to operate	Sugar conveyance cannot start		1 × 10^−3^ d^−1^ [31]	
		Fails to close	Rupture	High sugar flow, sugar may accumulate and spread in the environment		1 × 10^−4^ d^−1^ [31]	
1A–1B–4A (Aerobelt^®^ conveyor belts)	Conveyance of sugar	Low flow	Belt wearing	Sugar released inside the air cushion, formation of ignitable/explosive mixture		40 × 10^-6^ h^−1^ [5]	
		Fails to start, given run	No air availa-ble	Sugar conveyance cannot start, sugar accumulates at the outlet of the silo 3A		5 × 10^−2^ y^−1^ [31]	
1A–1B–4A (Aerobelt^®^ conveyors fans)	Conveyance of sugar	High air flow	Wrong voltage	High sugar flow, sugar may be dispersed and leak from the belt		1 × 10^−6^ h^−1^ [31]	Check for technical details
		Low air flow	Fan wearingORFan clogged	Low sugar flow		2 × 10^−6^ h^−1^ [5] 1 × 10^-6^ h^−1^ [31]	
4B (Roller conveyor belt)	Conveyance of sugar	Fails to start, given run	Electric circuit failure	Sugar conveyance cannot start, sugar accumulates at the outlet of the silo 3B–3C		1 × 10^−6^ h^−1^ [31]	
		Overheating	Roller wearing	Overheating can ignite eventual sugar dust deposits		5 × 10^−6^ h^−1^ [5]	
		Low flow	Belt wearing	Sugar may spread in the surroundings and accumulate inside rotating elements		40 × 10^-6^ h^−1^ [5]	
2A–2B (Bucket elevators)	Conveyance of sugar	Low flow	Roller wearing	Sugar conveyance cannot be performed regularly		5 × 10^-6^ h^−1^ [5]	
		Loose bucket	Bucket wearing	A falling steel bucket may generate a friction spark		Unlikely [30]	

**Table 6:** Human errors involved.

Operation	Human Error	Frequency
Sugar clogging	Operator omits procedure (“rodding”)	2.00 × 10^−3^ (-) [28]

**Table 7:** Process variables analyzed in the ROA Plus–ISD application.

Symbol	Process Variable
*F*	Sugar flow
*m*	Sugar mass
*C_a_*	Airborne sugar dust concentration
*T_m_*	Temperature of mechanical parts
*T_a_*	Air temperature
*P*	Pressure
*V*	Volume available

**Table 8:** Unavailability of components and human errors.

Component/ Human Error	Failure Mode	Failure Cause	Failure Rate (λ)	P_i_(t)	ID
4B Hand Switch	Load arc	Electric circuit wearing	Unlikely [30]	8.72 × 10^−3^	HSEl
	Fails to perform	Switch wearing	4.00 × 10^−6^ h^−1^	3.57 × 10^−2^	HSW
3A–3B–3C silos	Release of sugar	Vessel wearing	3.00 × 10^−5^ h^−1^	2.31 × 10^−1^	3AW–3BW–3CW
	Flow blocked	Sugar clogging	3.00 w^−1^	1.00	3AC–3BC–3CC
3A–3B–3C Discharge valves (motor driven)	Fails to open	Fails to operate	1.00 × 10^−3^ d^−1^	3.06 × 10^−1^	3AVO–3BVO–3CVO
	Fails to close	Rupture	1.00 × 10^−4^ d^−1^	3.58 × 10^−2^	3AVR–3BVR–3CVR
1A–1B–4A (Aerobelt^®^ conveyor belts)	Low flow	Belt wearing	4.00 × 10^−5^ h^−1^	2.96 × 10^−1^	1ABW–1BBW–4ABW
	Fails to start, given run	No air available	5.00 × 10^−2^ y^−1^	4.88 × 10^−2^	1ABNa–1BBWNa–4ABWNa
1A–1B–4A (Aerobelt^®^ conveyor fans)	High air flow	Wrong voltage	1.00 × 10^−6^ h^−1^	8.72 × 10^−3^	1ABWV–1BBWV–4ABWV
		Fan wearing	2.00 × 10^−6^ h^−1^	1.74 × 10^−2^	1ABFW–1BFW–4AFW
	Low air flow	Fan clogged	1.00 × 10^−6^ h^−1^	8.72 × 10^−3^	1AFC–1BFC–4AFC
4B (Roller conveyor belt)	Fails to start, given run	Electric circuit failure	1.00 × 10^−6^ h^−1^	8.72 × 10^−3^	4Bel
	Overheating	Roller bearings wearing	5.00 × 10^−6^ h^−1^	4.29 × 10^−2^	4BRW
	Low flow	Belt wearing	4.00 × 10^−5^ h^−1^	2.96 × 10^−1^	4BBW
2A–2B (bucket elevators)	Low flow	Roller wearing	5.00 × 10^−6^ h^−1^	4.29 × 10^−2^	2ARW–2BRW
	Loose bucket	Bucket wearing	1.00 × 10^−6^ h^−1^	8.72 × 10^−3^	2ABW–2ABW
“Rodding”	-	Operator omits procedure	-	2.00 × 10^−3^	HE
Friction spark	Fall of metal parts	-	Unlikely [30]	8.72 × 10^−3^	FrS
Enclosure	Enclosure	-	-	1.00-	5En

**Table 9:** Probabilities of occurrence P for every MCS identified. (MCS—Minimal Cut Sets)

Dust Cloud			Local Fire			Dust Explosion		
#	MCS	P(-)	#	MCS	P(-)	#	MCS	P(-)
1	3BVR,5En	3.58 × 10^−2^	1	3BVR,4BRW	1.53 × 10^−3^	1	3BVR,4BRW,5En	1.53 × 10^−3^
2	3CVR,5En	3.58 × 10^−2^	2	3BVR,HSEl	3.12 × 10^−4^	2	3BVR,HSEl,5En	3.12 × 10^−4^
3	4BEl,5En	8.71 × 10^−3^	3	3BVR,FrS	3.12 × 10^−4^	3	3BVR,FrS,5En	3.12 × 10^−4^
4	3BC,5En,HSW	3.57 × 10^−2^	4	3BW,4BRW	9.01 × 10^−3^	4	3CVR,4BRW,5En	1.53 × 10^−3^
5	3BC,5En,HE	2.00 × 10^−3^	5	3BW,HSEl	1.83 × 10^−3^	5	3CVR,HSEl,5En	3.12 × 10^−4^
6	3CC,5En,HSW	3.57 × 10^−2^	6	3BW,FrS	1.83 × 10^−3^	6	3CVR,FrS,5En	3.12 × 10^−4^
7	3CC,5En,HE	2.00 × 10^−3^	7	3CVR,4BRW	1.53 × 10^−3^	7	4BEl,4BRW,5En	3.74 × 10^−4^
			8	3CVR,HSEl	3.12 × 10^−4^	8	4BEl,HSEl,5En	7.60 × 10^−5^
			9	3CVR,FrS	3.12 × 10^−4^	9	4BEl,FrS,5En	7.60 × 10^−5^
			10	3CW,4BRW	9.01 × 10^−3^	10	3BC,4BRW,5En,HSW	1.53 × 10^−3^
			11	3CW,HSEl	1.83 × 10^−3^	11	3BC,4BRW,5En,HE	8.56 × 10^−5^
			12	3CW,FrS	1.83 × 10^−3^	12	3BC,HSEl,5En,HSW	3.11 × 10^−4^
			13	4BBW,4BRW	1.27 × 10^−3^	13	3BC,HSEl,5En,HE	1.74 × 10^−5^
			14	4BRW,4BEl	3.74 × 10^−4^	14	3BC,FrS,5En,HSW	3.11 × 10^−4^
			15	4BBW,HSEl	2.58 × 10^−4^	15	3BC,FrS,5En,HE	1.74 × 10^−5^
			16	4BBW,FrS	2.58 × 10^−4^	16	3CC,4BRW,5En,HSW	1.53 × 10^−3^
			17	4BEl,HSEl	7.60 × 10^−5^	17	3CC,4BRW,5En,HE	8.56 × 10^−5^
			18	4BEl,FrS	7.60 × 10^−5^	18	3CC,HSEl,5En,HSW	3.11 × 10^−4^
			19	3BC,4BRW,HSW	1.53 × 10^−3^	19	3CC,HSEl,5En,HE	1.74 × 10^−5^
			20	3BC,4BRW,HE	8.57 × 10^−5^	20	3CC,FrS,5En,HSW	3.11 × 10^−4^
			21	3BC,HSEl,HSW	3.12 × 10^−4^	21	3CC,FrS,5En,HE	1.74 × 10^−5^
			22	3BC,HSEl,HE	1.74 × 10^−5^			
			23	3BC,FrS,HSW	3.12 × 10^−4^			
			24	3BC,FrS,HE	1.74 × 10^−5^			
			25	3CC,4BRW,HSW	1.53 × 10^−3^			
			26	3CC,4BRW,HE	8.57 × 10^−5^			
			27	3CC,HSEl,HSW	3.12 × 10^−4^			
			28	3CC,HSEl,HE	1.74 × 10^−5^			
			29	3CC,FrS,HSW	3.12 × 10^−4^			
			30	3CC,FrS,HE	1.74 × 10^−5^			

## Appendix A

**Table A1:** ROA for the enclosed steel belt conveyor (Node 4B).

Rec	NDV	Causes	Consequences Due to Protections Failure	Plant State with Protections Working Correctly	Protections			Notes	TE
					Manual		Automatic Safety Systems Actions		
					Alarm (Optical/ Acoustic)	Operator Actions on Components			
4.1	4BhF	Discharge Valve 3B Rupture; OR Discharge Valve 3C Rupture	4Bhm		-	-	-	In case of a worn 4B belt sugar may slide into bearings	
4.2	4BlF	Sugar clogging 3B; OR Sugar clogging 3C ;OR 4B Belt fails to start (electrical circuit failure)	5hCa (due to Sugar transportation blocked) 4Bhm	System goes back to normal functionality	-	Belt Switch Off AND “Rodding” operation-		Provide operational procedures for cleaning lumps	TE1
4.3	4Bhm	4BhF; OR 4BlF; OR Wearing of 4B Belt; OR 3B vessel wearing; OR 3C vessel wearing	Sugar spillage		-	-	-	Sugar may slide into bearings, increasing their temperature and lowering their service life	TE2
4.4	4BhTm	Roller bearings wearing	4BhhTm		-	-	-	Bearings reach a temperature > LIT	
4.5	4BhhTm	4Bhm; AND (4BhTm; OR Friction spark; OR Electric spark)	5hTa (dust can be ignited by a hot spot or by a spark) Local fire					Install temperature sensor inside the enclosure close to the rollers	TE3

Rec—Record; TE—Top Event; h—high; hh—very high; l—low; T—temperature; P—pressure; C—concentration; F—flow.

**Table A2:** ROA for the enclosure (Node 5).

Rec	NDV	Causes	Consequences Due to Protections Failure	Plant State with Protections Working Correctly	Protections			Notes	TE
					Manual		Automatic Safety Systems Actions		
					Alarm (Optical/Acoustic)	Operator Actions on Components			
5.1	5hTa	4BhhTm	5hhTA					Combustion of sugar deposits triggers Temperature may reach values >MIT	
5.2	5hCa	(4BlF; OR 4BhF); AND 5LV	Sugar dust cloud		-	-		Require checking from operators, install dust collecting system	TE4
5.3	5hPa	5hCa; AND 5LV; AND (5hhTa; OR Friction spark; OR Electric spark)	Explosion					Install pressure sensor inside the enclosure	TE5
